# Investments in tuberculosis research – what are the gaps?

**DOI:** 10.1186/s12916-016-0644-0

**Published:** 2016-08-25

**Authors:** Mishal S. Khan, Helen Fletcher, London School of Hygiene and Tropical Medicine TB Centre Steering Committee, Richard Coker

**Affiliations:** 1 London School of Hygiene and Tropical Medicine, London, UK; 2 Saw Swee Hock School of Public Health, National University of Singapore, Tahir Foundation Building, 12 Science Drive 2 #10-01, Singapore, 117549 Singapore; 3 Faculty of Public Health, Mahidol University, Bangkok, Thailand

**Keywords:** Research agendas, Tuberculosis, Funding, Policy

## Abstract

Through decades of research, numerous studies have generated robust evidence about effective interventions for tuberculosis control. Yet, the global annual decline in incidence of approximately 1 % is evidence that current approaches and investment strategies are not sufficient. In this article, we assess recent tuberculosis research funding and discuss two critical gaps in funding and in scientific evidence from topics that have been left off the research priority agenda.

We first examine research and development funding goals in the 2011–2015 Global Plan to Stop Tuberculosis and analyze disbursements to different research areas by funders worldwide in 2014. We then summarize, through a compilation of published literature and consultation with 35 researchers across multiple disciplines in the London School of Hygiene and Tropical Medicine TB Centre, priorities identified by the tuberculosis research community. Finally, we compare researchers’ priority areas to the global funding agendas and activities.

Our analysis shows that, among the five key research areas defined in the 2011–2015 Global Plan - namely drugs, basic science, vaccines, diagnostics and operational research - drug discovery and basic science on *Mycobacterium tuberculosis* accounted for 60 % of the $2 billion annual funding target. None of the research areas received the recommended level of funding. Operational research, which had the lowest target, received 66 % of its target funding, whereas new diagnostics received only 19 %. Although many of the priority research questions identified by researchers fell within the Global Plan categories, our analysis highlights important areas that are not explicitly mentioned in the current plan. These priority research areas included improved understanding of tuberculosis transmission dynamics, the role of social protection and social determinants, and health systems and policy research.

While research priorities are increasingly important in light of the limited funding for tuberculosis, there is a risk that we neglect important research areas and encourage the formation of research silos. To ensure that funding priorities, researchers’ agendas and national tuberculosis control policies are better coordinated, there should be more, and wider, dialogue between stakeholders in high tuberculosis burden countries, researchers, international policymakers and funders.

**Electronic supplementary material:**

The online version of this article (doi:10.1186/s12916-016-0644-0) contains supplementary material, which is available to authorized users.

## Background

The ancient scourge, tuberculosis, was the subject of the world’s first randomized controlled trial reported in 1949 [1], and since then numerous studies have generated robust evidence about effective interventions for tuberculosis control [2]. Although highly effective treatment regimens have been around for many decades [3], in 2014, tuberculosis killed 1.5 million people, surpassing HIV to become the leading cause of death from an infectious disease globally [4]. Tuberculosis evades control efforts for numerous reasons, including the lack of timely access to quality diagnostic and treatment services for vulnerable populations, which has contributed to the spread of drug-resistant tuberculosis. At the current rate of decline in incidence – just over 1 % per annum – it will take more than 150 years to meet the World Health Organization (WHO) targets of reducing tuberculosis deaths by 95 % and incidence by 90 % compared to rates in 2015 [5].

Recognizing the need for major improvements in our progress on tuberculosis control, the Stop TB Partnership’s Global Plan to End TB 2016–2020 calls for a paradigm shift [5]. While an acknowledgement of the need for a change in approach is promising, the tuberculosis control community has been criticized for failing to act effectively on the basis of existing knowledge and for constantly looking for ‘new’ solutions. Through his analysis of responses to tuberculosis in the twentieth century, historian Christian McMillan highlights a pattern of ‘repetition and rediscovery’ among researchers and policymakers, owing to a tendency to ignore lessons that have been learnt [6], resulting in a squandering of resources on repeatedly addressing already answered research questions. This view is echoed in a review of numerous studies carried out by the British Medical Research Council’s tuberculosis units between 1946 and 1986, which made the striking assertion: “[by the late 1980s] *all of the measures necessary for successful programmes for the control of tuberculosis had been delineated*” [3]. On seeing the renewed calls for increased funding, some researchers have questioned whether we can justify being stewards of substantial funding for global health “*if we cannot manage a disease as well known as tuberculosis*” [7]. While the barriers to managing tuberculosis are numerous, including its association with poverty and the generation of drug resistance owing to inadequacies in health systems, these challenges are well defined; the balance between generating new knowledge and identifying strategies to implement proven solutions is thus being questioned.

In order to reflect upon, and learn from, our recent research activities and priorities, we look at the past 5 years of tuberculosis research funding and discuss two critical gaps in funding and in knowledge owing to essential topics being left off the research priority agenda.

## Methods

We first extracted data on the funding goals for key research areas in the 2011–2015 Global Plan to Stop TB (Global Plan). The total annual funding goal for 2014, in US dollars, was identified in the report for individual research areas according to the classification used by the authors (drugs, basic science, vaccines, diagnostics, operational research). Data on the amount of funding actually allocated by global funding bodies in 2014 was extracted from the 2015 Treatment Action Group report [2], classified into the same five research areas as used by the Global Plan. Using these data, we expressed the allocated funding amount for each research area as a percentage of the Global Plan funding goal to assess how much of the funding goal was achieved for each research area. We then followed a two-step process to qualitatively compile priority topics identified by tuberculosis researchers. In the first step, we obtained the full text of articles on tuberculosis research priorities published by numerous groups between 2006 and 2010 [8–13]; we ensured that articles covered a wide range of possible research areas within tuberculosis, including childhood tuberculosis, drug resistance, diagnostics, vaccines and HIV, as well as papers looking more broadly across the whole spectrum of tuberculosis control. We extracted, from text and tables in these articles, research topics that the authors identified as being important. We classified research topics into the same five research areas used in the Global Plan, or into ‘others’ if they did not fit into any of the five research areas. In the second step, we updated the list of researchers’ priority topics compiled based on the literature, with additional priority research questions identified through an in-person consultation (short, structured, one-to-one interviews conducted in November 2015) with 35 researchers across multiple disciplines in the London School of Hygiene and Tropical Medicine (LSHTM) TB Centre [14]. The interviews were conducted at the annual TB Centre meeting under the supervision of a PhD student at LSHTM, and were recorded with respondents’ permission. Respondents were asked to identify their top research priorities for tuberculosis control. One of the authors (MSK) transcribed the interviews verbatim, and classified priority research topics identified by researchers into one of the five Global Plan research areas or ‘others’. Duplication between priority research topics emerging from the literature and from analysis of interviews was removed, and those classified under ‘others’ were grouped based on recurring themes or topics. Finally, we combined the quantitative and qualitative data into a single table (Table 1) to compare researchers’ priority topics with the global funding agendas and activities, highlighting key gaps. The priority research topics that did not fit into one of the five Global Plan research areas (identified from analyzing common themes among topics classified as ‘other’ in the qualitative analysis) were summarized into a separate table (Table 2).

**Table 1:** Tuberculosis (TB) research priorities identified by researchers, funding received in 2014 and Global Plan funding targets for 2014

	2014 Global Funding ($m)		Priority areas identified by researchers^c^
	Target^a^	Actual spending^b^ (% of target)	
Drugs	740	243.3 (33)	Develop drugs (for drug-susceptible and drug-resistant TB) with higher potency, lower toxicity and shorter duration of treatment; new prophylactic regimens; host direct therapies
Basic science	420	150.1 (36)	Identify and validate biomarkers for monitoring decease activity, cure, relapse and of immune protection
Vaccines	380	111.3 (29)	Develop safe and effective vaccines (for adults and HIV-infected patients); understand variability in effectiveness
Diagnostics	340	65.4 (19)	Improve performance of existing tests; develop new point of care tests (for all forms, including latent and drug-resistant TB) that are cheap, rapid and sensitive
Operational research	80	52.8 (66)	Assess strategies to optimize implementation of new tools; to improve health worker performance, private provider engagement and integration of TB services

^a^2011–2015 Global Plan Target

^b^Treatment Action Group 2015 Report on Tuberculosis research Funding Trends, 2005–2014: A Decade of Data

^c^From literature and consultation with LSHTM TB Centre members

**Table 2:** Additional research priority areas that are not explicitly included in the Global Plan, as identified by researchers

Transmission dynamics	Which individuals are responsible for most tuberculosis transmission in high burden communities? How effective are different interventions in interrupting transmission?
Social determinants	How do structural and socioeconomic factors increase vulnerability to tuberculosis and how can they be addressed cost effectively?
Health systems and policy research	How can health systems be strengthened to better deliver quality services to at-risk populations (comorbidities, geographically isolated), thereby preventing generation of drug resistance? What measures should be taken to engage unregulated private healthcare providers? How do we increase evidence-based policy setting?

## Results

### Funding needs outlined in the Global Plan

The Global Plan to Stop TB 2006–2015 was launched in Davos, Switzerland, at the World Economic Forum in 2006 [15]. At $56 billion, the Stop TB Partnership’s forecasted total cost represented a three-fold increase in annual investment in tuberculosis control compared with the first Global Plan for 2001–2005 [16]. An update was provided for the 2011–2015 period in order to set out a clearer plan for reaching the Millennium Development Goals and Stop TB Partnership’s 2015 targets of halving tuberculosis prevalence and deaths compared with 1990 levels [16].

The research and development (R&D) component of the 2011–2015 Global Plan called for approximately $2 billion in annual funding to “*revolutionize the prevention, diagnosis and treatment of TB as the foundation for elimination of the disease*” [16]. Drug discovery and basic science were identified as the areas requiring the majority of investment (Table 1). With a target of $1.16 billion for 2014, these two areas accounted for 60 % of the recommended R&D funding. Basic science, which covers fundamental research about *Mycobacterium tuberculosis* and related organisms, was included as a separate research area in the updated plan, reflecting the fact that it underpins the development of all new technologies. The recommended level of R&D funding for basic science was set at $420 million per year. Similarly, operational research was included as a distinct research area in recognition of its essential role in ensuring uptake of new tools and efficient implementation of existing strategies. The funding allocated to operational research was, however, much lower than all other research areas, representing only 4 % of the 2014 target at $80 million.

### How much funding was available?

Research funding disbursed by public funding agencies, philanthropic and academic organizations and industry groups over the 2011–2015 period fell far short of the Global Plan goals. By the end of 2014, only $2.7 billion had been invested in tuberculosis R&D since 2011, just over one-fourth of the $9.8 billion called for. None of the research areas were funded at the target levels in 2014 (Table 1). Operational research met two-thirds of its target, higher than any of the other research areas, possibly because it had the lowest target. The greatest discrepancy between targeted and achieved funding was for new diagnostics, which received less than one-fifth of the $340 million goal for 2014.

To put tuberculosis research funding levels into context, an analysis of research investments for UK institutions concluded that tuberculosis is underfunded in comparison to HIV and malaria, despite causing the most mortality; between 2011 and 2013, tuberculosis research received only 20 % of the total $344 million funding, whereas HIV and malaria received approximately 40 % each [17]. Similarly, the Global Fund to Fight AIDS, Tuberculosis and Malaria, a funder that mainly provides programmatic support of which a small proportion goes towards research, allocated the lowest amount of funding to tuberculosis; in 2015, disbursements were $15.5 billion for HIV, $7.2 billion for malaria and $4.1 billion for tuberculosis [18].

### How well do research agendas match with researchers’ priority areas?

Within the individual research areas in the Global Plan, tuberculosis researchers’ priority objectives included developing better (more potent, less toxic, shorter duration of treatment) drugs for drug-resistant and drug-susceptible tuberculosis, identifying biomarkers for disease progression and immune responses, developing vaccines that are effective in adults and HIV-infected individuals, making accessible point of care diagnostics for all forms of tuberculosis and assessing strategies to optimize implementation of tuberculosis control strategies.

Additional priority research areas (not explicitly included in the Global Plan for R&D funding at present) were also highlighted by researchers. Improved understanding of tuberculosis transmission dynamics was identified as a key research need in order to plan more targeted, effective prevention interventions. The role of social protection and social determinants in tuberculosis is another area that researchers directed attention towards; indeed, there is a danger that neglecting to tackle socioeconomic determinants of tuberculosis may justify criticisms about failing to learn lessons from the massive reduction in tuberculosis incidence in much of Europe, which was achieved without drugs, vaccines or technologically-advanced diagnostics [19]. Finally, health systems and policy research is not yet included as an independent research area in the Global Plan. Health systems and policy research is distinct from operational research as the latter focuses on optimizing implementation of tuberculosis control tools and strategies, whereas the former is concerned with factors influencing policymakers’ decisions, integration of tuberculosis control programs within the wider health system, and studies to inform optimal allocation of resources for tuberculosis control. Funding of both operational and health systems and policy research is essential for achieving maximum impact on tuberculosis control with limited resources, an aspect that the tuberculosis control community is currently struggling with. While most researchers cited priority research questions that were linked to their own work, those working on development of new drugs, vaccines and diagnostics recognized the importance of research to ensure that new tools can be accessed by tuberculosis patients in resource limited settings.

## Discussion

Funding priorities are essential for the effective allocation of limited resources and they can act as a focal point for driving financial investment. However, it is important to critically consider funding priorities that are being set and who is setting them. Policymakers and funders often prefer a biomedical approach to disease control and pay less attention to addressing more complex sociopolitical realities and their impact on the causal pathways of disease [20]. It is thus encouraging that the new Global Plan to End TB 2016–2020 recognizes that “*medical interventions alone will not be enough to end tuberculosis*” and stresses the importance of Universal Health Coverage and social determinants [5]. It is now vital to ensure that the R&D component of the Global Plan – which currently focuses on only three biomedical components: drugs, vaccines and diagnostics – prioritizes funding for research on health systems strengthening, translation of research into effective policies, addressing social determinants of tuberculosis, understanding transmission hotspots and analyses which inform optimal resource allocation.

We must also recognize that some funding streams may encourage researchers to restrict their activities to discrete silos, separating applied or implementation research from ‘hypothesis driven’ biomedical studies. We should therefore ensure greater support for cross-discipline research, which many funders are now emphasizing; for example, to explore the influence of poverty on immune correlates and to assess the impact of different investments (new drugs or vaccines, versus socioeconomic improvements versus Universal Health Coverage) on reductions in tuberculosis transmission and incidence.

Finally, there has to be flexibility in funding of priority areas identified by the research community and national tuberculosis programs themselves, such as health systems and policy research and studies on socioeconomic determinants, allowing researchers to be proactive and not only reactive to specific funding calls. Here, tuberculosis researchers must engage with funders and global policymakers to ensure that the research findings inform global tuberculosis control efforts more effectively. A pragmatic solution would be to support the formation of national bodies for setting research agendas, including key national stakeholders and researchers engaged in the country, which inform global funding priorities and play a role in evaluating research proposals in terms of applicability and potential impact on tuberculosis in high burden settings. Engaging local researchers would not only build research capacity and reduce costs from engaging primarily international teams, but also potentially aid dissemination and uptake of findings into policy.

Resource limitations did not allow us to expand the scope of our interviews to include researchers and policymakers in high tuberculosis burden countries; such a study would allow an important additional comparison of research priorities in international versus national institutions. Furthermore, we believe that a simple exercise in which national tuberculosis program representatives score the potential impact of findings from a number of recently completed studies would be very informative, and potentially surprising, for researchers and funders alike. Finally, interviews with representatives of key funding agencies would help to better understand why some diseases are funded disproportionately relative to disease burden (or potential public health impact), why narrow technology or biomedical approaches are often prioritized and how best to influence the research funding agenda.

## Conclusions

There appears to be some disconnect between funding priorities, researchers’ agendas and global disease control strategies. Tuberculosis receives much less research funding than HIV and malaria despite causing more deaths globally, and the available funding is often channeled towards biomedical approaches; the 2011–2015 Global Plan recommended that far more funding be allocated to basic science and drug discovery than operational research to maximize the impact of new tools and, although the new Global Plan to End TB 2016–2020 highlights that biomedical interventions alone are not sufficient, the R&D component still focuses on drugs, vaccines and diagnostics. Improved coordination between stakeholders in high tuberculosis burden countries, researchers, international policymakers and funders would help to ensure that critical funding and knowledge gaps are addressed, and existing knowledge is acted upon more effectively.

## Additional file

Additional file 1: Response to reviewers. (DOCX 120 kb)
