# Supplementary material for: Investments in tuberculosis research – what are the gaps?
Source: BMC Med. 2016 Aug 25;14(1):123. doi: 10.1186/s12916-016-0644-0 (PMC4997680; doi:10.1186/s12916-016-0644-0)
Supplement: Additional file 1: — Response to reviewers. (DOCX 120 kb) [file 12916_2016_644_MOESM1_ESM.docx]

9 May 2016

Dear Paulina,

Thank you very much for arranging peer review of our paper and for confirming that you prefer submission as a correspondence.

The reviewers’ comments are encouraging and constructive, particularly as both reviewers noted that this is an important/flagship article. We have addressed all the comments in our revised version of the paper and our responses are detailed below. As requested, we have included an unstructured abstract.

With the minor edits that have certainly strengthened the paper, I hope it is now suitable for publication. I look forward to hearing from you.

Best Regards,

Mishal Khan

1. I am a little confused by the format and structure of the paper. It is listed as correspondence, but has a structured abstract, background and methods section, and then proceeds to present some results and some discussion but not in their own sections and instead across a range of sub-headings. There may well have been previous communications between journal and authors about how to present the paper, but to me, it felt a little confusing to read the paper as it is. I would suggest it would be better to present this work as a traditional 'original article', with intro/methods/results/discussion. That in turn leads on to…
**RESPONSE – Since the journal prefers submission as a correspondence we have included an unstructured abstract for consistency.**

2. There seems an excellent opportunity in the discussion to further emphasise some key points about R&D funding, and key areas in tuberculosis. There is some limited consideration, but I was left feeling that the paper could (without a huge amount of extra effort) cover so much more and be a flagship publication for people who have and need to have these sorts of discussions (funders, policymakers and as the authors point out, researchers). It may be that as a correspondence piece, word counts etc are limited but if so, I would encourage both journal and authors in rethinking that. Further thinking on this are below.
**RESPONSE – We agree with your suggestions and have added to the discussion**

Specific points (using the line numbers shown in the pdf)

Throughout - why abbreviate tuberculosis to TB? It doesn't save on word count, and I assume character count is not an issue.

**RESPONSE – We have changed to tuberculosis throughout as suggested.**

Abstract/background - 'On World TB Day'. Actually on World TB Day that you did this? Or 'for World TB Day'?

**RESPONSE – The paper was written to be part of the World TB Day supplement, but since this has now passed we have removed the reference to World TB Day.**

Abstract/background - 'discuss two critical gaps…'. No need to mention 'gap' twice thereafter in that sentence, you've already highlighted that.

**RESPONSE – Thank you; we have edited this.**

Background, line 66 - 'many many'. Suggest losing a 'many', it doesn't add the desired extra emphasis and feels a little casual.

**RESPONSE – Edited as suggested.**

Background, line 66-67. Reference to TB surpassing HIV in numbers of deaths. I'd like to see this quantified, what are the numbers involved, what's the scale of the problem?

**RESPONSE – TB killed 1.5 million people in 2014, compared to 1.2 million from HIV. We have added the TB death toll to the sentence identified by the reviewer.**

Background, line 67 - 'It is clear that…'. I agree with the assertion approaches have been insufficient, but this is at odds with two sentences previously when you stated that highly effective treatments have been around for decades. Suggest slight revisions or clarifications for consistency. Perhaps also a sentence on AMR at this point, and/or other reasons as to why these seemingly successful treatments have not been effective in control and reductions in burden.

**RESPONSE – We have made slight revisions as suggested**

Aim of this paper, line 90-91. Again, excess use of 'the gap'

**RESPONSE – Thank you; we have edited this.**

Methods, general point - more detail please. The reader would not be able to replicate your approaches. **RESPONSE – We have added more detail in this section.**

Specifically on this -
Line 96 - 'and assessed…'. How did you assess? What method or analytical approach? Or was it simply using information collected by TAG (in which case 'considered' might be a better word than 'assessed')
Line 98 - 'we summarised'. How?

**RESPONSE – We have provided more detail on the process followed, replacing the word summarised.**

Line 100-103. This consultation - how was it done? Semi-structured questionnaire? Interviews? Written responses? Focus group?

**RESPONSE – We conducted short in person interviews (one-one) and have added some detail in the paper.**
Line 103 - 'we mapped'. Again, how? In itself, 'mapped' doesn't mean too much outside of the geographer's office, so what did you actually do here?

**RESPONSE – We have replaced the word ‘map’ with ‘compare’ for clarity**

Line 110 - 'At $56 billion…'. This is in a paragraph headed 'funding needs'. So is this number the suggested need, or amount actually allocated. Would also be good to clarify here if this number is public and philanthropic, or includes private sector investment (and is thus an estimate of 'everything')

**RESPONSE – We have clarified that this is the total funding required as estimated by the WHO.**

Line 135 - 'As illustrated in figure 1'. Firstly, isn't it a table, rather than a figure? Secondly, suggest deleting that bit and adding 'figure/table 1' in brackets at the end of the sentence, after 'levels in 2014'.

**RESPONSE – Edited as suggested**

Line 139 - '19%'. There is a mixture of use of fractions and percentages, suggest choosing one approach and being consistent with it.

**RESPONSE – We have edited to use only fractions.**

Line 141 - 'an analysis of UK funding'. Note of pedantry, it was an analysis to UK institutions, rather than 'UK funding' per se. Also, for added justification as to why the levels of funding matter, same analysis also showed investment for malaria and HIV better than for TB when compared to various global burden of disease metrics - [http://www.ebiomedicine.com/article/S2352-3964(15)30244-9/fulltext](http://www.ebiomedicine.com/article/S2352-3964%2815%2930244-9/fulltext)

**RESPONSE – We have clarified and added some additional detail based on the reviewer’s comments**

Line 142 - ' was a distribution very unevenly'. Correct this.
**RESPONSE – Thank you; done**

Line 145-147. Latter part of sentence doesn't read very clearly, suggest '$7.2 billion for malaria and $4.1 billion for TB'

**RESPONSE – We have edited based on the reviewer’s comments**

Line 158 - 'were also highlighted'. By who, is this still the researcher's views and considerations here?

**RESPONSE – Yes; we have clarified that this is reflecting researchers’ views**

Line 171 - by the end of this paragraph,  I'm wondering what topics were not considered to be priority areas, this list seems to shout "prioritise everything!", which in itself isn't so helpful.
Was there any attempt to tease out which areas were most often mentioned as top priority? If not, then perhaps mention that no one really agreed on what were the top 3 or 5 most important topics, and list the results from the qualitative work in a table/appendix?

**RESPONSE – There were some research areas that were not mentioned as key priorities (ie. vaccines for adults was prioritised over more research on vaccine for children; low cost point of care diagnostics were prioritised over high tech, expensive ones), so we mention only the specific priority research areas highlighted by researchers in table 1 and in the new table 2. We did not do a group exercise which would have allowed ranking of priority areas, so have mentioned that most researchers highlighted priority areas linked to their research focus, but that there was a consistent recognition of the importance of research that will allow operational and policy impacts.**

Line 173 - sub-heading. 'we'. Who is 'we'? Researchers, funders, policymakers, private sector, all of the above?

**RESPONSE – This subheading has been removed to indicate the start of the discussion**

Line 187-188, 'discrete silos' - Need to justify why you think that, I would argue back that the funding landscape is increasingly prescribing investment for cross-disciplinary research consortia; I'm also not sure what  you mean by 'structure of current funding priorities'

**RESPONSE – We have changed to use the term ‘funding streams’ instead of ‘structure of current funding priorities’ and added a comment to balance the argument that some funders are calling for cross-disciplinary research**

Line 193, ''has to be flexibility'. Again here, if I were a funder, I would argue that there already is huge flexibility, as many calls are 'anything at all', sent to an infection and immunity programme at MRC, Wellcome et al. So need to justify this statement (e.g. Euro Commission are often top down with little bottom-up funding, Gates are mostly quite prescriptive about what they want to see etc)

**RESPONSE – Here we meant flexibility on which research areas should be prioritised for funding (ie. not flexibility on specific studies within infection and immunity, but rather for researchers to be able to propose studies on health systems functioning if they feel this is more important for TB control than more biomedical approaches). We have provided some examples to clarify our statement.**

For further discussion pointers - If you are to flesh out the discussion, I'd point towards there not being enough mention of
- need for R&D to tackle AMR
- co-morbidities with diabetes and other areas
- economic costs of TB and AMR & TB not terribly well known.

**RESPONSE – We have added an additional table fleshing out some of the research areas and specific questions. In this we mention AMR and co-morbidities among other topics.**

Plus also, if you are going to be critical of how research investments are prioritised and disbursed, what improvements would the authors suggest? (You mention this a little, but it can be easily rebutted at the moment; this is a chance prescribe something with impact). What new or novel funding sources could there be to tackle the gaps (the mooted Global Health Research Fund, the Ross Fund set up in the UK etc etc). Who are they key stakeholders who must be motivated to make that impact? What the authors pragmatic recommendations about ways forward?

**RESPONSE – Thank you for these suggestions; we have added some pragmatic recommendations on page 10.**

And so what that TB gets less funding than HIV or malaria - so does smallpox, but that's okay isn't it? (A deliberately silly example, but you get the point, why is this important?).

**RESPONSE – We have emphasised (on page 7) that the reason for concern over low TB funding relative to other infectious diseases is that it causes the most deaths; funding is thus not reflective of disease burden.**

Also, requires a focus on limitations of the methods (e.g. interviewed only researchers from one institution, large though the group is, plus no funders interviews who would no doubt have different viewpoints, as might other stakeholders). One presumes that this being unfunded work there were also resource constraints on those involved. And so on.
**RESPONSE – We have added a paragraph on page 10 addressing limitations and areas for further study.**

Reviewer #3: Investment in tuberculosis research - what are the gaps?
This is an interesting assessment of TB research spending and recognized gaps.  Overall the manuscript is well written and should be of general interest.  There are no major reservations, just minor issues/corrections for consideration.
**RESPONSE – We appreciate the supportive comments.**

The comment on p7 "…some researchers have questioned whether we can justify being stewards of substantial funding for global health "if we cannot manage a disease as well known as TB" (7) " presents a gross over-simplification of the issue (albeit attributed to "some researchers".  It would be better if this comment is more nuanced and acknowledges some of the intrinsic challenges - such as the strong link between poverty/inequality and TB - something that remains a massive global challenge, with TB essentially serving as a marker of our failure to eradicate poverty.  Also the long co-evolution of M.tb and H. sapiens (with an intricate immune balance being established), which distinguishes TB from most infectious diseases that have been successfully eliminated or greatly reduced
**RESPONSE – We agree and have added some text about the challenges of controlling TB.**

Methods
Correct to read: We first compiled data…"
**RESPONSE – This is done.**

"for key areas in the 2011-2015 Global Plan to End TB" - This must be incorrect; the End TB strategy was only launched in 2015

**RESPONSE – We have corrected the report name to ‘Global Plan to Stop TB’.**

P6 - "…, was included as a separate research area in the updated plan, …"
How much funding was available?

**RESPONSE – We have added that $420 million was the recommended funding level for basic science.**

P7 "…- equivalent to $2 billion - was distributed very unevenly…"

**RESPONSE – This paragraph has been edited for clarity**

Priority areas
P7 Correct to read: "..(more portent, less toxic, shorter duration treatment)…
**RESPONSE – Our intended meaning was more potent ie. powerful drugs**

P8 - Why is improved understanding of TB transmission dynamics of value/interest ? - to guide better targeted public/global health responses

**RESPONSE – Yes, to guide more effective strategies for prevention. We have added some words to clarify.**

Table
Drugs - What about Host Directed Therapy (see recent 2016 Lancet ID review)?

**RESPONSE – We have added this to the table**
Basic Science - What about Understanding the disease process/natural history of primary and re-infection events?  Evolution/Co-evolution of M.tb and humans?
Vaccines - What about better vaccines for vulnerable young children? - has this been completely abandoned?
Diagnostics - What about a marker of re-infection after cure or treatment for latent infection?  Marker for incipient disease in someone known to be infected?
**RESPONSE – As suggested by reviewer 2, we include only the key priority areas identified to avoid putting down everything that may be important. This is not to say that they areas excluded are of no interest.**

Would be good to add the suggested new funding areas at the bottom - indicating priority areas within the new funding areas identified - any suggested funding targets?

**RESPONSE – We have added an additional table with new funding areas and specific research questions**

In summary, this is a solid paper that adds interesting new information, but it will be improved if minor corrections are made and a few areas better clarified.
**RESPONSE – Thank you; we have addressed all the useful comments you provided.**


Comments from Guest Editors

1. Line 102 refers to LSTHM as ‘the largest collection of researchers…..’ I think this is a unnecessary claim and open to challenge (who have they counted, what qualifies them to be included, etc) and actually could lead to the view that analysis responses from one institution with  a fairly narrow remit is of reduced relevance.

**RESPONSE – Thank you; we have removed this statement.**
